# Clinical characteristics and functional improvement of patients admitted to a Child and Adolescent Autism Spectrum Disorders (ASD) Day Therapeutic Unit

**DOI:** 10.1192/j.eurpsy.2023.356

**Published:** 2023-07-19

**Authors:** A. Alvarez, N. Santamaria, V. Bote, B. Sanchez, J. A. Monreal, A. Hervas

**Affiliations:** ^1^Hospital Universitario Mutua Terrassa, Terrassa; ^2^Hospital Universitario Mutua Terrassa, Terrassa, Spain

## Abstract

**Introduction:**

The ASD Day Therapeutic Unit of the HUMT is an interdisciplinary reference center specialized in ASD, for the care of children and adolescents with this pathology, that offers care by programs with the aim of achieving functionality altered.

**Objectives:**

The main objective of this study is to know the clinical characteristics of the patients admitted to our center and to offer preliminary data on the functional improvement achieved in a pilot program that works by processes.

**Methods:**

This is a prospective clinical study of patients with ASD, treated at the ASD Day Therapeutic Unit of the HUMT since februay 2022 till nowadays. We compare the clinical improvement and functionality acquired through the evaluation through various scales: Conners scale, SRS, BRIEF and CBCL.

**Results:**

Our sample is made up of 19 patients with ASD who are admitted to the HUMT ASD Day Hospital. 13 belonged to the intensive care program, 5 to recovery of low-functioning functionality, and 1 to the differential diagnosis program. 84.21% (n=16) have preserved cognitive capacity. The median age is 13.1 years. 73.7% of the sample are men.

We observed that 89% of patients admitted to our unit presented comorbid symptoms with ADHD. more than 78.9% present isolation, anxiety/depression, altered thinking and attention difficulties. 13/19 present a SEVERE RANGE OF AFFECTATION upon admission, in social area. Facing discharge, the CBCL shows us a general improvement in behavior and comorbidity in the total scale.

The SRS shows us an improvement in social awareness, social communication, social motivation, repetitive behavior and stereotypes and in the total score.

**Conclusions:**

Specific interventions in social skills, autonomy, social understanding, daily routines, and sensory integration in patients with ASD improve core symptoms, as well as associated behavior.

Specific interventions and working on social relationships during admission improve comorbidity derived from ASD.

More studies are needed to specify the most efficient interventions to improve the quality of life in children and teenagers with ASD.

**Disclosure of Interest:**

None Declared

